# Coronary cross-sectional area stenosis severity determined using coronary CT highly correlated with coronary functional flow reserve: a pilot study

**DOI:** 10.1038/s41598-025-11920-z

**Published:** 2025-07-23

**Authors:** Takuto Koumoto, Shozo Kusachi, Takumi Tomiya, Takuya Akagi, Hiroshi Kawamura, Satoshi Hirohata, Hirosuke Yamaji, Takashi Murakami, Shigeshi Kamikawa, Masaaki Murakami

**Affiliations:** 1 Division of Radiation, Okayama Heart Clinic, Takeda 54-1, Naka-Ku, Okayama, 703-8251 Japan; 2 Division of Cardiovascular Intervention, Okayama Heart Clinic, Takeda 54-1, Naka-Ku, Okayama, 703-8251 Japan; 3 Division of Cardiovascular Medicine, Okayama Heart Clinic, Takeda 54-1, Naka-Ku, Okayama, 703-8251 Japan; 4 Heart Rhythm Center, Okayama Heart Clinic, Takeda 54-1, Naka-Ku, Okayama, 703-8251 Japan; 5 Okayama University Graduate School of Health Sciences, 2-5-1 Shikata-cho, Kita-ku, Okayama, 700-8558 Japan

**Keywords:** Ischemic heart disease, Reversible ischemia, Coronary pressure, Multi-slice CT, Coronary hemodynamics, Cardiology, Medical research

## Abstract

Fractional flow reserve (FFR) is the gold standard for assessing the physiological significance of coronary stenosis. We examined the potential correlation between digitally measured coronary cross-sectional area stenosis using coronary computed tomography (CT) angiography and FFR. We analyzed data of 32 consecutive patients with stenoses who underwent invasive FFR determination. The cross-sectional area was assessed using 128-slice coronary detector-based spectral CT angiography. Power analysis revealed that the sample size enabled the detection of an area under the receiver operating characteristic (ROC) curve (AUC) of 0.90. FFR ≤ 0.8 and > 0.8 were defined as FFR-positive and FFR-negative, respectively. Intra- and interobserver differences were negligible. Percentage cross-sectional area stenosis was calculated as 100 × (A−B)/A, where A is the cross-sectional area at non-stenotic pre-stenotic segment and B is the cross-sectional area of the most severe stenotic lesion. AUC indicated that percentage cross-sectional area stenosis effectively discriminated between FFR-positive and FFR-negative cases, yielding a sensitivity of 0.882 and specificity of 0.933 at a cutoff of 50% area reduction, with an AUC of 0.976. Lesions with less than 45% cross-sectional area stenosis on coronary CT angiography were not FFR-positive. When ROC analysis was conducted for lesion characteristics, AUC did not significantly improve. In conclusion, the percent coronary cross-sectional area stenosis measured using coronary CT angiography distinguished between FFR-positive and FFR-negative lesions with high accuracy. The severity of coronary cross-sectional area stenosis determined using CT angiography is clinically useful for predicting FFR.

## Introduction

Percutaneous coronary intervention (PCI) is now widely used for treating coronary artery disease, particularly ischemic heart disease.^1,2^ Decisions regarding PCI require an evidence of reversible ischemia and examination of the extent of area perfused by the coronary artery distal to the culprit stenotic lesion. The relationship between coronary stenosis severity and ischemia has been extensively examined. A decrease in hyperemic flow after coronary occlusion has been demonstrated to reduce coronary flow reserve. This diminution in flow is most evident at hyperemic states and begins as early as 40% narrowing of vessel diameter, with more predictable reductions in hyperemic flow for stenoses of ≥ 70%.^3^ Another study reported that among patients with 70% stenosis, only 32% exhibited severe ischemia and 40% manifested no or mild ischemia according to myocardial perfusion scintigraphy.^4^

Coronary luminal diameter is usually measured visually or using the caliper method; however, these methods are not accurate. Furthermore, coronary vessel diameter stenosis does not accurately reflect the severity of coronary stenosis. Coronary stenosis is complicated as it is not concentric and is usually eccentric to some degree. The relationship between the grade of coronary diameter stenosis and myocardial ischemia is complex, with ensuing studies demonstrating an unreliable relationship between coronaly diamter stenosis and ischemia.^5^ Previous studies did not demonstrate that diameter reduction is a reliable marker for reversible ischemia.

On the other hand, fractional flow reserve (FFR) is used for determining reversible ischemia caused by the responsible stenotic lesion. FFR is an index of the physiological significance of coronary stenosis and is defined as the ratio of maximal blood flow in a stenotic artery to normal maximal flow.^6^ FFR is the ratio of distal coronary pressure, measured using a coronary pressure guidewire, to aortic pressure, measured simultaneously using the guiding catheter. The process of measuring FFR is easy. The FFR of a normal coronary artery is 1.0, and an FFR value of 0.80 or less indicates ischemia-causing coronary stenoses with an accuracy of more than 90%.^6–8^ Thus, FFR is considered the gold standard for determining physiologically significant coronary stenosis. However, the method used for measuring FFR is invasive and requires intracoronary pressure measurement. To estimate FFR non-invasively, a fluid dynamics model applied to coronary CT angiography has been attempted.^9^ The model involves many hypotheses and employs complex equations.^10^ The fluid dynamic model has its own limitations.

Based on these considerations, we refocused on the morphological analysis of coronary stenosis. We hypothesized that the severity of coronary cross-sectional area stenosis may correlate with FFR. The only one study that examined the relationship between cross-sectional area stenosis and FFR reported a theoretically untenable poor relationship. The study focused on plaque volume, and different CT systems were used. No study has used detector-based spectral CT for evaluating cross-sectional area stenosis, which can provide high-quality images with sufficient resolution power. Therefore, our study focused on analyzing the association of cross-sectional area stenosis severity and stenosis characteristics with FFR using 128-slice detector-based spectral CT.

## Methods

### Patients

This study was conducted at Okayama Heart Clinic. We analyzed data of 32 consecutive patients with stable coronary heart disease who underwent coronary CT and FFR measurements using coronary angiography. Informed consent was obtained from all patients. An informed consent form outlined the objectives, advantages, disadvantages, and safety concerns of the study.

The required minimal sample size was > 9 FFR-positive lesions and > 9 FFR-negative lesions to detect an area under the receiver operating characteristic (ROC) curve (AUC) of 0.90, with a power of 0.90 and a significance level of α = 0.05 in an ROC analysis.^11^.

All examinations and analytical procedures adhered to the principles of the Declaration of Helsinki 2000, and the study was approved by the Institutional Ethics Committee for Human　Research of the Okayama Heart Clinic (approval number, TK1). Written informed consent for the use of data without personally identifiable information was obtained from all patients.

### Fractional flow reserve measurements

Coronary angiography was performed using a coronary angiography catheter (Good Tec HT™, Goodman, Nagoya, Japan) and a vascular introducer (Radifocus IIH, Terumo, Tokyo, Japan). FFR was defined as the ratio between the distal coronary pressure and aortic pressure, both measured simultaneously during maximal coronary hyperemia according to established methods.^8^ Coronary pressure was measured using a coronary pressure guidewire (OmniWire; Philips Japan, Tokyo, Japan). Maximal hyperemia was induced by a bolus administration of nicorandil (4 mg) into the intracoronary artery. Pullback coronary pressure recordings were performed to discriminate between focal and diffuse disease. FFR was calculated by dividing the mean distal coronary pressure by the mean aortic pressure during hyperemia. The FFR was considered diagnostic of ischemia at a threshold of 0.80 or less.^10^.

### Coronary computed tomography scan protocol and cross-sectional area measurement

Coronary CT angiography was performed using a 128-slice detector-based spectral CT system (Brilliance iCT SP; Philips Healthcare, Cleveland, OH, USA) and an automatic dual-head injector (Stellant DualFlow; Nihon MEDRAD K.K., Osaka, Japan). All patients received 0.6 mg of nitroglycerin spray (Myocor™, Astellas Pharma, Tokyo, Japan) and 2–8 mg of propranolol (Inderal; AstraZeneca, London, UK) administered intravenously 5 min before the examination to reduce heart rate when it exceeded 70 beats per minute in the absence of contraindications. Contrast medium (Iopamidol™ 370 mg iodine/mL; Bayer Yakuhin, Osaka, Japan) was intravenously administered at 4.5 mL/s depending on body weight (0.7 mL/kg), followed by 20% diluted contrast medium (30 mL). Imaging was initiated using a bolus-tracking method. The position of the reconstruction window within the cardiac cycle was individually optimized to minimize motion artifacts. Axial images of 0.8-mm slice thickness were reconstructed at 0.4-mm intervals using a multi-cycle reconstruction algorithm with a medium-smooth cardiac kernel (XCB). Coronary CT angiography was used to evaluate the alignment of the coronary arteries between slabs of data acquired during consecutive heart cycles. Images exhibiting coronary vessel discontinuity due to motion or arrhythmia were excluded from the study.

**Fig. 1:**
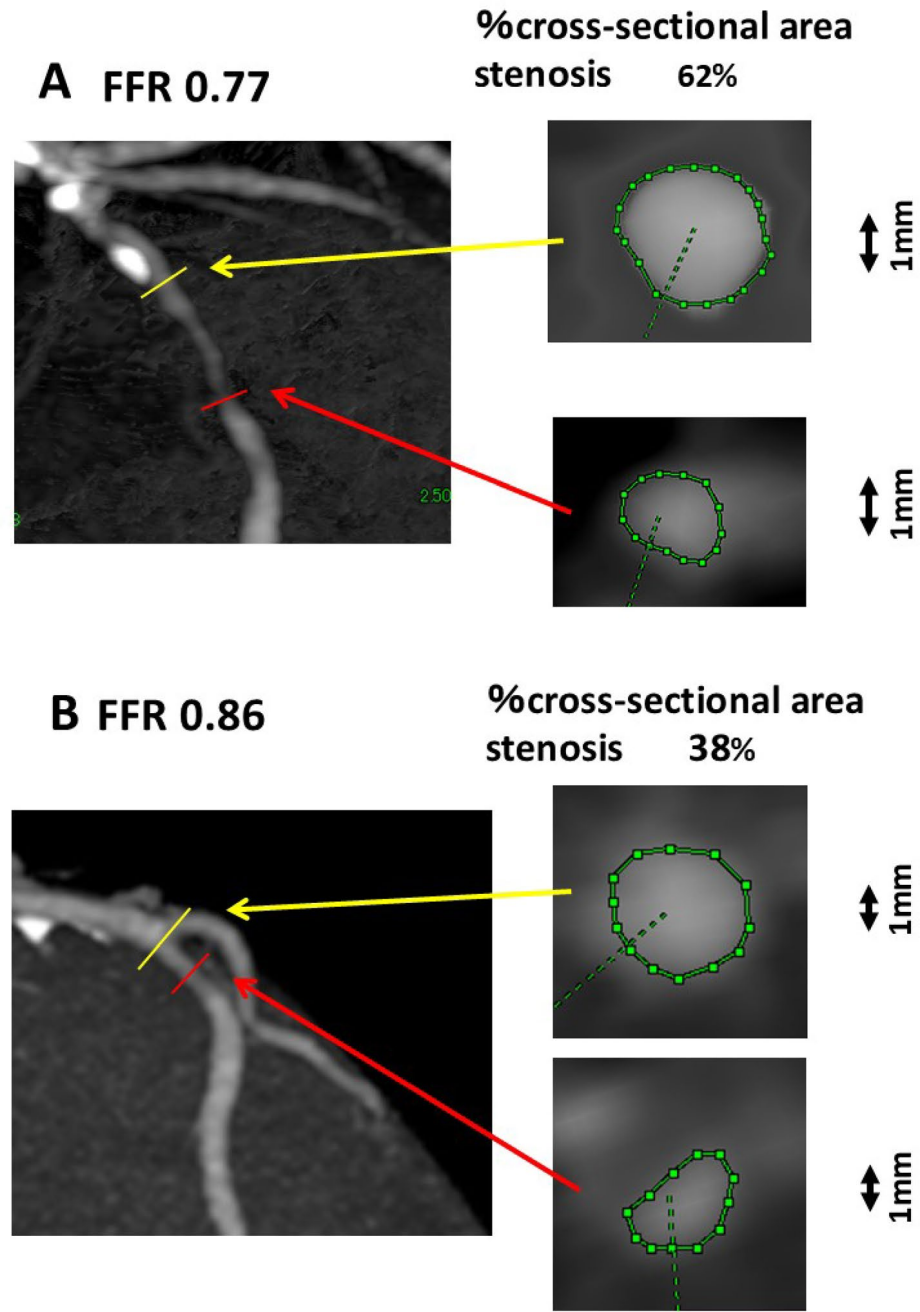
CT angiography images and cross-sectional views of a non-stenotic pre-stenosis segment (yellow arrow) and of the most stenotic segment (red arrow) are shown for two patients: one with a positive FFR (upper panel, A) and one with a negative FFR (lower panel, B). CT, computed tomography; FFR, functional flow reserve.

Coronary CT was analyzed using a CT workstation (Extended Brilliance Workspace; Philips Healthcare, Cleveland, OH, USA). Figure 1 shows the coronary CT angiography images, including a cross-sectional image of a non-stenotic pre-stenosis segment and an image at the segment with the most severe stenosis in two patients: one with a positive and one with a negative FFRs. The cross-sectional area of the coronary artery was measured by tracing it on a high-resolution display with a pen. Inter- and intraobserver differences in coronary cross-sectional area measurements were checked in 10 randomly selected areas of stenotic and non-stenotic lesions. The percentage of cross-sectional area stenosis was calculated as follows: 100 × (A − B)/A, where A is the cross-sectional area at non-stenotic pre-stenosis segment, and B represents the cross-sectional area of the most severe stenotic lesion. The cross-sectional area stenosis was graded as follows: score 0, ≤ 40% stenosis; score 1, > 40% and ≤ 50% stenosis; score 2, > 50% and ≤ 60% stenosis; and score 3, ≥ 60% stenosis. Both the actual and scored values of the percentage cross-sectional stenosis severity were used for statistical analysis.

### Examination of characteristics of other lesions

In addition to cross-sectional area stenosis, the correlation of length and irregularity of stenotic lesions, irregularity of pre-stenotic lesions, and calcification of the lesion with FFR were examined. Irregularities were graded from 0 to 2. Calcification was graded from 0 to 4. Grading was conducted by referring to sample images of each grade, and when two observers did not agree on the grade, an additional observer checked, and grading was finalized. The actual length of the stenotic lesion was graded as follows: score 0, ≤ 10 mm; score 1, > 10 and ≤ 20 mm; score 3, > 20 and ≤ 40 mm; and score 5, > 40 mm.

### Statistical analysis

Statistical analyses were performed using R version 3.2.2 (R Foundation for Statistical Computing, Vienna, Austria).^12^ The power analysis for paired comparison, correlation analysis, and determination of AUC in ROC curve was conducted using G*Power 3.1.9.7.^11^ Inter- and intraobserver differences in the measurements of coronary cross-sectional area were evaluated using linear regression analysis and Bland–Altman plots. A Student’s t-test or Mann–Whitney U-test was used to compare the data between patients with an FFR of ≤ 0.80 (FFR-positive group) and those with an FFR of > 0.80 groups (FFR-negative group) in accordance with the data distribution pattern. The Kolmogorov–Smirnov test and histograms were used to determine whether the data were normally distributed. The homogeneity of variance was checked using the F-test. Fisher’s exact test with 2 × 2 or m × n tables and two-tailed tests for categorical variables were used to compare the two groups or more than three groups when appropriate. Correlation analysis was performed to identify factors, including percentage coronary cross-sectional area stenosis, that demonstrated correlation with FFR. Simple and multivariate regression analyses were conducted to evaluate the relationship between these factors and FFR. ROC curve analysis was used to assess the discriminatory power of these factors, including percentage cross-sectional area stenosis, in distinguishing between FFR-positive and FFR-negative lesions. The AUC and optimum cutoff level were determined using the ROC curve analysis to evaluate the discriminant power ability. Although the number of patients was small and multivariate analysis may have demonstrated a low accuracy, an ROC curve using propensity scores derived from multiple logistic regression analysis was used as a reference. Before the ROC analysis, multiple linear regression analysis was performed with actual FFR values as independent variables to evaluate the correlation of the factors with FFR values. Based on the results, the scores were selected for use in the ROC analysis. Data are presented as mean ± one standard deviation (SD) or as median with 25th and 75th percentiles. Statistical significance was set at *p* < 0.05.

## Results

### Inter- and intraobserver differences

The correlation analysis revealed a correlation coefficient of 0.99 for both intra- and interobserver differences. The linear regression analysis revealed a regression coefficient and intercept of 0.98 and 0.42, respectively, for intraobserver differences. Similarly, a regression coefficient and intercept of 0.96 and 0.08, respectively, were obtained for interobserver differences. The Bland–Altman plots showed good agreement between the first and second observations and between one and the other examiner’s measurements. In both intra- and interobserver differences, all differences between the two measurements were located within 1.96 SD of the differences in the Bland–Altman plots.

### Patients’ clinical characteristics

Table 1 summarizes the results of the comparison of patient clinical characteristics between the FFR-positive and FFR-negative groups. No significant differences in clinical characteristics were observed between the two groups.

**Table 1:** Patients’ clinical characteristics.

		Total	FFR-positive	FFR-negative	*p*-value
Number of patients		32	17	15	
Age	years	72 ± 9	69 ± 9	75 ± 8	0.100
Female	n (%)	7 (24)	6 (35)	1 (7)	0.088
Body mass index	kg/m^2^	24.1 ± 3.6	24.5 ± 3.5	23.8 ± 3.7	0.585
Hypertension	n (%)	21 (66)	9 (52)	10 (65)	0.362
Diabetes mellitus	n (%)	9 (28)	6 (35)	3 (20)	0.197
Hyperlipidemia	n (%)	19 (59)	10 (59)	9 (60)	0.535
Creatinine clearance	mL/min	89 ± 28	91 ± 28	80 ± 21	0.455

FFR, functional flow reserve.

### Univariate analysis for angiographic measurements

Table 2 summarizes the results of angiographic measurements. The FFR-positive group showed a significantly lower percentage of coronary cross-sectional area stenosis, indicating a higher degree of stenosis than that in the FFR-negative group. The length of stenosis tended to be longer in the FFR-positive group than in the FFR-negative group.

**Table 2:** Comparison between FFR-positive and FFR-negative groups.

	Total	FFR-negative	FFR-positive	*p*-value
Number of patients	32	15	17	
FFR	0.830 ± 0.090	0.901 ± 0.044	0.749 ± 0.048	< 0.001
Percentage cross-area stenosis (%)	44 ± 19	30 ± 13	60 ± 11	< 0.001
Stenosis lesion length (mm)	17.1 ± 11.1	14.0 ± 89	20.7 ± 12.4	0.088
Irregularity of stenotic lesion; grades 0 : 1 : 2	7 : 19 : 6	1 : 10 : 4	6 : 9 : 2	0.142
Irregularity of pre-stenotic lesion; grades 0 : 1 : 2	16 : 15 : 1	7 : 7 : 1	9 : 8 : 0	0.854
Calcification of stenotic lesion; grades 0 : 1 : 2 : 3 : 4	11 : 10 : 6 : 4 : 1	7 : 3 : 3 : 2 :0	4 : 7 : 3 : 2 : 1	0.502

Correlation analysis	Correlation coefficient		*p*-value	
FFR vs. percentage cross-sectional area stenosis	−0.811		< 0.001	
FFR vs. stenosis lesion length	−0.340		0.057	
FFR vs. irregularity of stenotic lesion	−0.385		0.029	
FFR vs. irregularity of pre-stenotic lesion	−0.244		0.178	
FFR vs. calcification of the lesion	−0.085		0.643	

FFR, functional flow reserve.

The simple correlation analysis revealed that the percentage coronary cross-sectional area stenosis and grades of irregularity of stenosis significantly correlated with FFR. Other angiographic factors did not correlate with the actual FFR values.

### Multivariate analysis for angiographic measurements

The results of the multiple regression analysis, with the actual FFR values as the independent variable and angiographic factors as the dependent variables, are presented in Table 3. Among the factors examined, only percentage coronary cross-sectional area stenosis demonstrated a significant correlation with FFR.

**Table 3:** Results of multiple linear regression analysis.

Independent variable						
Actual FFR value	Partial regression coefficient	SE of partial regression coefficient	Standardized regression coefficient	Partial correlation coefficient	F value	*p*-value
*Dependent variables*						
% cross-sectional area stenosis	− 0.0036	0.0005	− 0.7635	− 0.8025	47.0300	< 0.001
Length of stenotic lesion	− 0.0001	0.0010	− 0.0166	− 0.0272	0.0190	0.8907
Irregularity of stenotic lesion	− 0.0219	0.0233	− 0.1582	− 0.1816	0.8870	0.3558
Irregularity of stenosis origin	− 0.0331	0.1911	− 0.2095	− 0.3215	2.9970	0.0953
Calcification of the lesion	0.0096	0.0110	0.1235	0.1694	0.7680	0.3887
Constant	1.0220					
Multiple correlation coefficient: 0.7928, *p* < 0.001						

FFR, functional flow reserve; SE, standard error.

### Receiver operating characteristic curve analysis

**Fig. 2:**
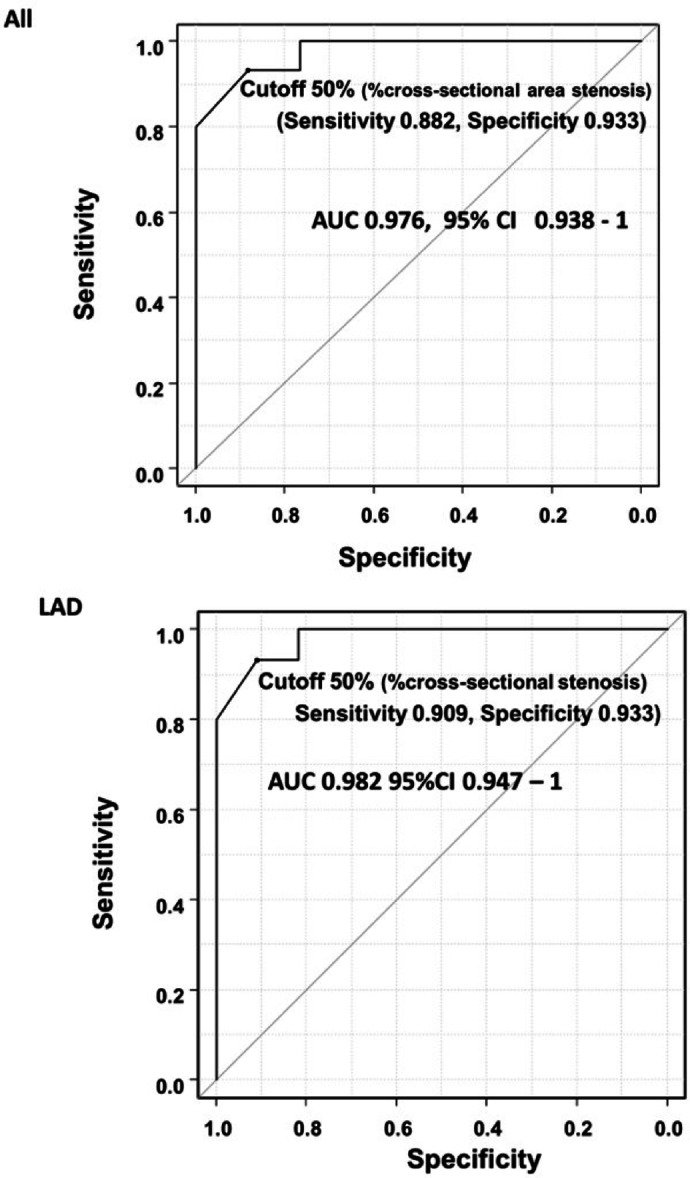
ROC curve analysis for determining the severity of coronary cross-sectional area stenosis to distinguish FFR-positive from FFR-negative stenosis. The upper panel shows all stenoses, and the lower panel shows stenoses located in the LAD. AUC, area under the curve; LAD, left anterior descending coronary artery; FFR, functional flow reserve; CI, confidence interaval.

Figure 2 illustrates the ROC curve analysis conducted to distinguish between the FFR-positive and FFR-negative groups. The power analysis showed that detecting an AUC of 0.95 in the ROC analysis required a sample size of > 9 in the FFR-positive and FFR-negative groups to obtain a statistical power of 0.95, with α = 0.01. Therefore, the number of FFR-positive and FFR-negative patients was sufficient to detect an AUC of 0.95. The AUC results are presented in Fig. 2 and summarized in Table 4. The results demonstrated that percentage cross-sectional area stenosis showed high accuracy for distinguishing FFR-positive patients from FFR-negative patients with a sensitivity of 88% and specificity of 93% at a cutoff value of 50%. When the analysis was performed only for left anterior descending artery lesions, an identical or slightly superior AUC value was obtained. Additionally, ROC analyses of the length and irregularity of stenosis, in addition to percentage cross-sectional area stenosis, were performed using propensity scores derived from the multiple logistic regression analysis. The results did not show a significant increase in AUC compared with that obtained with percentage cross-sectional area stenosis.

**Table 4:** Results of receiver operating characteristic curve analysis for distinguishing between FFR-positive and FFP-negative groups.

Patients	Number of patients	Applied factor	Scores or actual value	AUC	95% confidence interval	Cutoff value	Sensitivity	Specificity
All patients	n = 32	% cross-sectional area stenosis	actual value	0.976	0.938–1.000	50%	0.882	0.933
Patients with LAD lesions	n = 26	% cross-sectional area stenosis	actual value	0.982	0.947–1.000	50%	0.909	0.933
All patients	n = 32	% cross-sectional area stenosis	scored value	0.927	0.837–1.000	Score 2	0.882	0.933
Patients with LAD lesions	n = 26	% cross-sectional area stenosis	scored value	0.936	0.845–1.000	Score 2	0.909	0.933
All patients	n = 32	% cross-sectional area stenosis and stenosis length and irregularity	PS	0.980	0.939–1.000	PS 0.713	1.000	0.933
Patients with LAD lesions	n = 26	% cross-sectional area stenosis and stenosis length and irregularity	PS	0.982	0.942–1.000	PS 0.785	1.000	0.933

AUC, area under the curve; FFR, functional flow reserve; LAD, left anterior descending coronary artery; PS, propensity score.

## Discussion

The major findings of the present study were as follows: (1) the severity of cross-sectional area stenosis, as measured using coronary CT, significantly correlated with FFR, and (2) the severity of cross-sectional area stenosis demonstrated a high AUC, effectively distinguishing FFR-positive patients from FFR-negative patients with high accuracy.

Significant relationships between the grades of morphological coronary stenosis and coronary flow reserves have been well demonstrated experimentally in dogs.^13,14^ Several clinical studies have evaluated the effects of the severity of coronary stenosis on coronary flow reserve. One study examined coronary flow reserve using digital subtraction angiography, where the area of the coronary cross-section was determined based on the square of the coronary diameter, instead of the planimetry tracing method. The study found significant relationships between the grade of coronary area stenosis and impairment of flow reserve, which indicated the reversibility of ischemia.^15^ A coronary are stenosis of 50–70% was associated with a decrease in flow reserve, whereas coronary are stenosis > 70% severely decreased flow reserve. Another study examined coronary dimensional stenosis and exercise testing for the detection of reversible myocardial ischemia in 276 patients. Coronary stenosis was evaluated using a combination of simple visual assessment, caliper measurements, and digital measurements. This study found that a 75% luminal diameter stenosis is the best cutoff point for determining significant coronary stenosis.^16^ A coronary diameter stenosis of 70–80%, associated with an AUC of 0.7–0.8, has been reported to distinguish a positive exercise test from a negative one. These results are in good agreement with the present study’s results, although the target index used in that study was a positive exercise test, while our study focused on FFR-positive lesions. These results support the fact that the grade of coronary stenosis plays an essential role in the impairment of coronary flow reserve, which causes reversible myocardial ischemia.

Among several methods available for detecting significant coronary stenosis, including exercise testing, digital subtraction angiography, positron emission tomography, and FFR, FFR has been demonstrated to be a reliable method for detecting significant pathophysiological coronary stenosis.^6,8,17^ FFR is now widely used as the gold standard for detecting significant lesion-specific coronary stenosis. FFR-guided PCI exhibits better clinical outcomes, including a reduction in death, nonfatal myocardial infarction, and repeat revascularization.^18^ Thus, the use of FFR as a reliable index of significant coronary stenosis was appropriate for this study.

Our study found a strong correlation between the severity of coronary cross-sectional area stenosis and FFR. In contrast, coronary artery luminal diameter stenosis showed a weak correlation with FFR. A previous study measuring FFR using invasive coronary angiography and coronary luminal diameter stenosis using coronary CT angiography (54-slice or dual-source) found that luminal diameter stenosis did not correlate well with FFR.^19^ Similarly, another study visually determined coronary luminal diameter stenosis in 1414 lesions and reported that while coronary diameter showed some correlation with FFR, it was not accurate, with FFR.^5^ Their findings revealed that coronary luminal diameter stenosis was unreliable in predicting FFR.

To the best of our knowledge, only one study had examined coronary area stenosis using coronary CT angiography and lesion ischemia using FFR.^20^ The authors reported a poor AUC of 0.66 for distinguishing lesions associated with ischemia from those without ischemia. The reasons for the difference in AUC results were unclear. The study was performed in two centers, using dual-source CT (Somatom Definition, Siemens, Forchheim, Germany) or 320-detector row CT (Aquilion One, Toshiba, Otawara, Japan). In contrast, the present study was performed in a single center, using a 128-slice detector-based spectral CT. These differences might have accounted for the different results regarding AUC. Despite this, many studies have reported significant relationships between luminal diameter stenosis and reversible ischemia. Although coronary luminal diameter stenosis did not strongly correlate with reversible coronary ischemia, a study found that an AUC of 0.7–0.8 and a cutoff point of 75% luminal diameter reduction may help detect reversible ischemia, as supported by a stress test. Thus, the previously reported AUC of 0.66 from coronary area stenosis was considerably lower than established AUCs for diameter reduction. In contrast, our AUC findings are consistent with those of previous studies.^15,16^.

Regarding the estimation of flow reserve at the site of coronary stenotic lesions using coronary CT angiography, a computational flow kinetics model has been developed to predict FFR.^9,10^ A study analyzed 159 vessels using computational flow dynamics and reported an AUC of 0.90 for distinguishing FFR-positive from FFR-negative lesions.^21^ Another study found a slightly lower AUC of 0.81 for the determination of FFR-positive lesions.^22^ Accuracy, sensitivity, and specificity for discrimination of FFR of ≤ 0.80 from that of > 0.80 were reported to be 73%, 90%, and 54% respectively.^22^ However, the model involves many hypotheses and uses complicated equations, limiting its applications for estimating FFR. Our study’s multiple linear regression analysis revealed that other factors, such as irregularity and calcification of stenotic lesions, did not correlate with FFR or improve AUC, indicating that the severity of coronary cross-sectional area stenosis is a major factor related to FFR.

The present limitations of the present study are as follows: First, although the power analysis indicated that the numbers of FFR-positive and FFR-negative patients included were sufficient for detecting an AUC of 0.95, further studies with a larger sample size are warranted to confirm the validity of the present findings and to identify the predictors of discrepancies between cross-sectional area stenosis and FFR. In addition, the numbers of right coronary artery (RCA) and left circumflex artery (LCx) lesions were small. Statistically, the findings for RCA and LCx were not outliers, but an increase in the number of RCA and LCx lesions would further confirm the validity of our results. Third, the present study did not analyze complex bifurcation lesions. Studies on these lesions will build upon the findings of the present study.

In conclusion, the severity of coronary cross-sectional stenosis determined digitally using coronary CT angiography can distinguish FFR-positive from FFR-negative lesions at a cutoff value of 50% coronary cross-sectional area stenosis with a high sensitivity (0.882) and specificity (0.933) in non-complex stenosis.

## Data Availability

Data supporting the findings of this study are available from the corresponding author, T. Ko., upon reasonable request.
